# Inflammatory Biomarkers Associated with In-Hospital Mortality in Critical COVID-19 Patients

**DOI:** 10.3390/ijms231810423

**Published:** 2022-09-09

**Authors:** Krisztina Pál, Anca Alexandra Molnar, Adina Huțanu, János Szederjesi, Ionuț Branea, Ágota Timár, Minodora Dobreanu

**Affiliations:** 1 Department of Laboratory Medicine, Emergency Clinical County Hospital, 540136 Targu Mures, Romania; 2 M2 Department, George Emil Palade University of Medicine, Pharmacy, Science, and Technology of Targu Mures, 540142 Targu Mures, Romania; 3 Department of Laboratory Medicine, George Emil Palade University of Medicine, Pharmacy, Science, and Technology of Targu Mures, 540142 Targu Mures, Romania; 4 Department of Anesthesiology and Intensive Care, George Emil Palade University of Medicine, Pharmacy, Science, and Technology of Targu Mures, 540142 Targu Mures, Romania; 5 Department of Anesthesiology and Intensive Care, Emergency Clinical County Hospital, 540136 Targu Mures, Romania; 6 Center for Advanced Medical and Pharmaceutical Research, George Emil Palade University of Medicine, Pharmacy, Science, and Technology of Targu Mures, 540136 Targu Mures, Romania

**Keywords:** IL-6, inflammation, COVID-19

## Abstract

The COVID-19 pandemic poses global healthcare challenges due to its unpredictable clinical course. The aim of this study is to identify inflammatory biomarkers and other routine laboratory parameters associated with in-hospital mortality in critical COVID-19 patients. We performed a retrospective observational study on 117 critical COVID-19 patients. Following descriptive statistical analysis of the survivor and non-survivor groups, optimal cut-off levels for the statistically significant parameters were determined using the ROC method, and the corresponding Kaplan-Meier survival curves were calculated. The inflammatory parameters that present statistically significant differences between survivors and non-survivors are IL-6 (*p* = 0.0004, cut-off = 27.68 pg/mL), CRP (*p* = 0.027, cut-off = 68.15 mg/L) and IL-6/Ly ratio (*p* = 0.0003, cut-off = 50.39). Additionally, other statistically significant markers are creatinine (*p* = 0.031, cut-off = 0.83 mg/dL), urea (*p* = 0.0002, cut-off = 55.85 mg/dL), AST (*p* = 0.0209, cut-off = 44.15 U/L), INR (*p* = 0.0055, cut-off = 1.075), WBC (*p* = 0.0223, cut-off = 11.68 × 10^9^/L) and pH (*p* = 0.0055, cut-off = 7.455). A survival analysis demonstrated significantly higher in-hospital mortality rates of patients with values of IL-6, IL-6/Ly, AST, INR, and pH exceeding previously mentioned thresholds. In our study, IL-6 and IL-6/Ly have a predictive value for the mortality of critically-ill patients diagnosed with COVID-19. The integration of these parameters with AST, INR and pH could contribute to a prognostic score for the risk stratification of critical patients, reducing healthcare costs and facilitating clinical decision-making.

## 1. Introduction

The outbreak of the COVID-19 pandemic caused by infection with the recently identified SARS-CoV-2 virus determined unprecedented pressure on healthcare systems worldwide. Intensive care management of critically ill patients poses enormous challenges given the often unpredictable clinical evolution observed in this category of patients.

Reported mortality rates in critical patients admitted to the intensive care unit (ICU) are high, varying between 12.9% and 61.5% among different centers [1,2,3]. However, despite the considerable mortality rate associated with severe COVID-19 cases, thus far there is no widely accepted and validated prognostic system for prediction of in-hospital mortality.

The hyperinflammatory syndrome, arising as a consequence of a dysregulated host immune response, represents a cornerstone of COVID-19 pathogenesis [4,5,6]. In the development of the cytokine storm, characterized by an excessive production and release of inflammatory mediators, a key part is played by interleukin-6 (IL-6), a pleiotropic inflammatory cytokine [7,8]. Moreover, dynamic variations of IL-6 have been associated with the survival of severe COVID-19 patients admitted to the ICU [9]. IL-6 has been proposed as a predictive biomarker for disease severity, in-hospital mortality, the necessity of intensive care and invasive mechanical ventilation [10,11]. C-reactive protein (CRP) is an acute phase reactant with a transcription in hepatocytes regulated primarily by IL-6 [12]. Higher levels of CRP on admission have been linked to disease progression to a severe form, indicating the potential utility of CRP as an early predictive biomarker for COVID-19 severity [13,14]. An additional acute phase protein thought to be correlated with disease severity and ICU mortality is ferritin [15,16]. Increased D-dimers reflect a hypercoagulable status and may be an indirect manifestation of COVID-19 inflammation [17].

A cost-effective and feasible suggested method of investigating the need for invasive mechanical ventilation and mortality in COVID-19 patients includes the calculation of inflammatory indices such as neutrophil to lymphocyte ratio (NLR) and systemic immune inflammation index (SII), defined as platelet count*neutrophil count/lymphocyte count [18,19]. Other potentially relevant complete blood count-derived biomarkers to predict COVID-19 severity are the platelet to lymphocyte ratio (PLR) and the monocyte to lymphocyte ratio (MLR) [20,21]. The interleukin-6 to lymphocyte ratio (IL-6/Ly) is a promising newly investigated immune-inflammatory index that could be used for the early risk stratification of COVID-19 patients [22]

The primary aim of our study consisted of identifying inflammatory laboratory markers associated with the in-hospital mortality in critical COVID-19 patients. Our secondary aim was to identify other routine laboratory parameters with predictive value on the survival status of critical patients.

## 2. Results

### 2.1. Clinical Characteristics of Patients

The study included 117 critical COVID-19 patients meeting the established inclusion criteria. Out of this total, a number of 89 patients (76.06%) died during hospitalization. A comparison of the clinical characteristics between the two groups (survivors vs. non-survivors) is shown in Table 1. There were no statistically significant differences regarding the median duration of hospitalization between survivors and non-survivors (9.5 respectively 10 days, *p* = 0.3744). In the non-survivor group, the mean age was significantly higher (66 ± 12 years) compared to the mean age of survivors (58 ± 14 years) (*p* = 0.0033). In the non-survivor group, 46 patients (51.68%) were of the male gender, while in the survivor group, 20 patients (71.42%) were male. Administration of anti-inflammatory therapies with tocilizumab and antiviral therapy with remdesivir were not significantly different between the two groups. The presence of more than one comorbid condition (excluding type 2 diabetes, which was analyzed separately) had similar frequencies in the two groups. Type 2 diabetes as a comorbidity present at admission demonstrated no significant differences between the two groups. Bacterial pulmonary infections as complications arising in critical COVID-19 patients were not significantly associated with survival status during hospitalization, according to our analysis.

The laboratory parameters for our study population are presented in Table 2. Out of the inflammatory biomarkers, IL-6 (*p* = 0.0004) and CRP (*p* = 0.027) demonstrated significantly higher values in the non-survivor group. On the other hand, there appeared to be no significant difference in the values of ferritin and D-dimers among the two groups. The IL-6/Ly ratio represented the only inflammatory index with significantly elevated values in the non-survivor group (*p* = 0.0003). Significantly higher levels of AST (*p* = 0.0209), creatinine (*p* = 0.0306) and urea (*p* = 0.0002) were encountered in the non-survivor group. Regarding the coagulation function, INR values were higher in non-survivors (*p* = 0.0055). The only acid-base parameter with significant differences between the two groups was pH, having lower values in non-survivors (*p* = 0.0055). Analysis of the CBC parameters indicated significantly higher WBC counts in non-survivors (*p* = 0.0223).

### 2.2. Establishing Optimal Cut-Off Levels for the Statistically Significant Biomarkers

**Figure 1:**
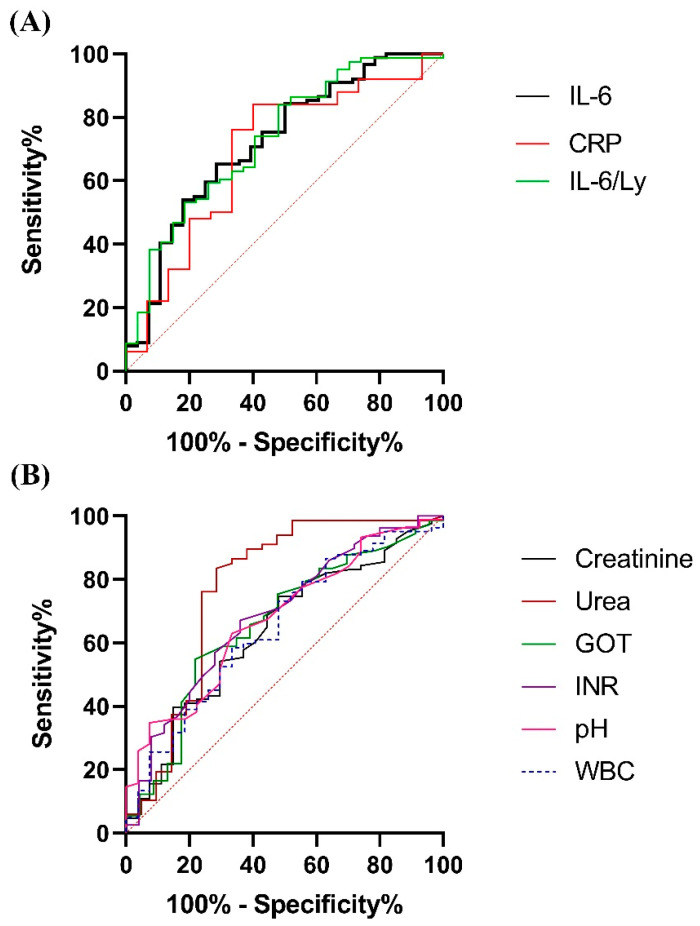
ROC curves for (**A**) inflammatory biomarkers (IL-6, CRP, IL-6/Ly); (**B**) routine laboratory parameters (creatinine, urea, AST, INR, pH and WBC).

We evaluated the prognostic accuracy of the laboratory parameters that presented significant differences between survivors and non-survivors and established their respective optimal cut-off levels, by using the ROC curve method. Statistically significant values were observed for IL-6, CRP, IL-6/Ly ratio, creatinine, urea, AST, INR, pH and WBC; ROC curves for these parameters are found in Figure 1.

The cut-off levels for each of these parameters and the corresponding AUCs are shown in Table 3. For the interpretation of AUCs, we considered values over 0.7 as denoting good accuracy and between 0.6–0.7 as meaning sufficient accuracy. All the investigated markers had at least sufficient predictive accuracy.

### 2.3. Survival Analysis

Survival data analysis for all parameters with statistically significant differences between survivor and non-survivor patient groups is summarized in Table 4.

**Figure 2:**
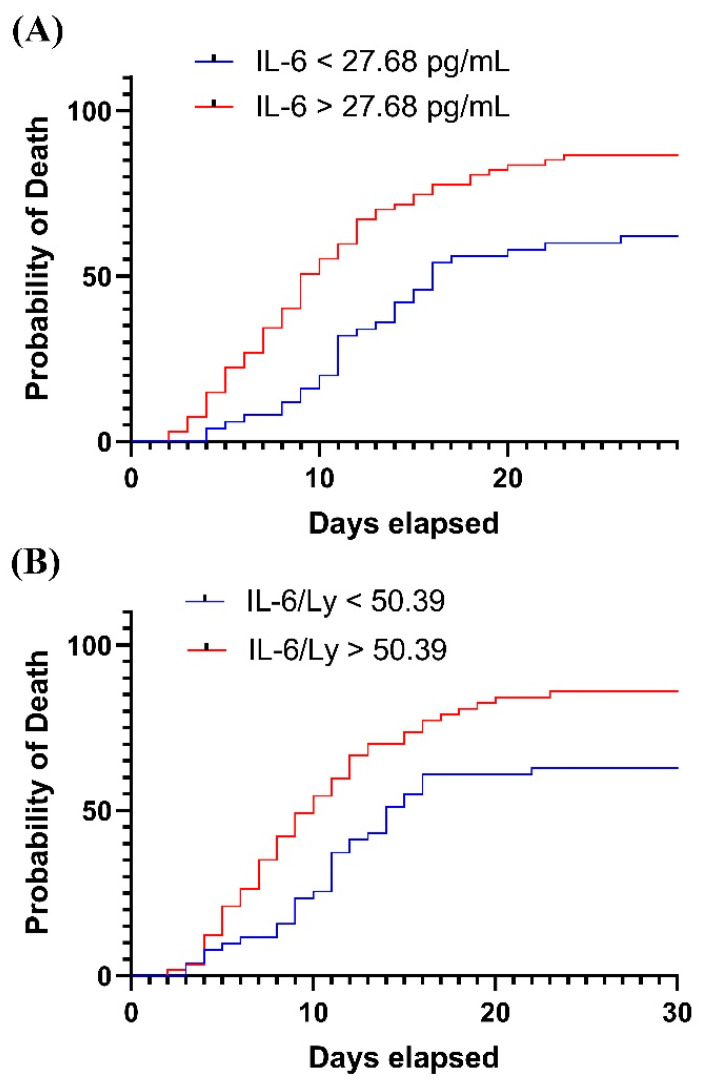
Kaplan-Meier curves for (**A**) IL-6; (**B**) IL-6/Ly.

Analysis of survival data by the Kaplan-Meier method indicated significantly higher mortality rates for patients with elevated IL-6 (>27.68 pg/mL, *p* = 0.0002) and an increased IL-6/Ly ratio (>50.39, *p* = 0.0019). As shown in Figure 2, the probability of death was double after 10 days of hospitalization for patients with IL-6 or IL-6/Ly values that exceeded the mentioned thresholds.

**Figure 3:**
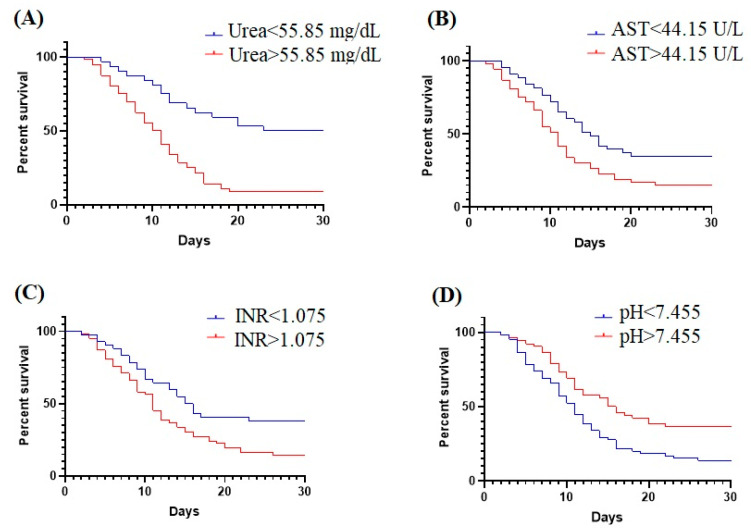
Kaplan-Meier curves for (**A**) urea; (**B**) AST; (**C**) INR; (**D**) pH.

Significantly lower survival rates were observed for patients with increased urea (>55.85 mg/dL, *p* < 0.0001), AST (>44.15 U/L, *p* = 0.0209), INR (>1.075, *p* = 0.0091). In addition, decreased pH (<7.455, *p* = 0.0029) is associated with a lower survival rate for critical patients (Figure 3).

No significant changes in survival rates were registered in patients with elevated CRP, creatinine or WBC counts.

## 3. Discussion

Two years after COVID-19 was declared a global public health emergency, the coronavirus illness still raises constant difficulties in patient care. These ongoing medical challenges are not only due to the emergence of novel viral variants leading to successive waves of infection, but also to the variable, hard to predict clinical course of disease, particularly in critically ill patients. The progression of the SARS-CoV-2 infection to severe manifestations such as acute respiratory distress syndrome, multiple organ failure or even fatal complications has hyperinflammation as a pathogenic basis [23].

In this context, establishing a panel of routine laboratory tests to guide the risk stratification of patients would prove highly beneficial to ensure both an optimal clinical management and judicious allocation of medical resources. In our study, we investigated the prognostic value of inflammatory biomarkers and of routine laboratory parameters on the in-hospital mortality of critically ill COVID-19 patients. Our analysis suggests that inflammatory markers such as the IL-6, CRP and IL-6/Ly ratio could potentially be used as predictors of in-hospital mortality for critical COVID-19 patients. Moreover, we found other routinely available laboratory tests (AST, creatinine, urea, WBC, INR and pH) with a possible prognostic value on the survival status of patients diagnosed with SARS-CoV-2 infection admitted to the ICU. These findings are laying a foundation in the perspective of developing a prognostic score system for risk stratification of critical COVID-19 patients.

We observed significantly elevated values of IL-6 (*p* = 0.0004) and CRP (*p* = 0.0270) in the non-survivor group. Our findings are consistent with medical literature data, and several other studies reported higher IL-6 levels in severe COVID-19 cases [24,25,26,27] and higher CRP in correlation with disease severity or adverse outcomes [11,27,28]. In a meta-analysis of 6320 patients, increased IL-6 and CRP were associated with a higher probability of mortality [29]. A retrospective analysis of critical COVID-19 patients, admitted to the ICU, identified IL-6 as an independent predictor of mortality [30], while a cohort study of critically ill SARS-CoV-2 patients found baseline high levels of IL-6 and CRP in association with both disease worsening and 60-day mortality [31]. Brasen et al. demonstrated that concentrations of SARS-CoV-2 RNA and antigen in the blood correlate with inflammation, IL-6 levels and mortality [32].

Based on the ROC curve analysis we performed, IL-6 (AUC = 0.721) as well as the IL-6/Ly ratio (AUC = 0.731), have a superior predictive accuracy when compared to CRP, which only demonstrates a sufficient predictive accuracy (AUC = 0.689). Although a number of studies indicate the predictive role of IL-6 and CRP, there is substantial heterogeneity between the suggested cut-off points for these parameters. In our study, we found cut-off values of 27.68 pg/mL (sensitivity 65.17%; specificity 67.86%) for IL-6 and 68.15 mg/L (sensitivity 76%; specificity 66.67%) for CRP. A prospective cohort study identified cut-off values of 86 pg/mL for IL-6 and of 87.5 mg/L for CRP [33]. Another retrospective study of inflammatory biomarker levels at admission found cut-off values of 74.98 pg/mL and of 81 mg/L for IL-6 and CRP, respectively [34]. Similar IL-6 cut-off values to the ones in our analysis used for predicting fatal outcome were reported by Zhou et al. [35] in a retrospective study of 66 patients, out of which only 17 were critical cases. A retrospective cohort study of 140 patients, including a number of 33 severe patients, finds cut-off levels of 32.1 pg/mL for IL-6 and 41.8 mg/L for CRP in regard to the development of complications, not mortality [36]. In a prospective cohort study comparing the prognostic role of inflammatory markers in COVID-19 and other respiratory infections, IL-6 and CRP are reported as the strongest predictors for ICU admission or death, at 30 days. [37] However, in this analysis, critical patients are not considered separately. By contrast to our results, a study conducted in the ICU of a rural hospital in India demonstrated no association between IL-6 and clinical outcomes [38]. This contradictory finding highlights the need for larger, multicentric studies investigating the predictive value of inflammatory biomarkers in critical COVID-19 patients.

The composed immune-inflammatory index IL-6/Ly ratio was significantly higher in the non-survivor group (*p* = 0.0003), with a cut-off point of 50.39 (sensitivity 60.49%; specificity 70.37%) and AUC = 0.731. In our analysis, the statistical power of IL-6/Ly ratio is given solely by IL-6, with the lymphocyte count demonstrating no significant difference between patient groups. Masotti et al. [39] report the prognostic value of IL-6/Ly ratio in COVID-19 pneumonia for a combined endpoint of in-hospital mortality and/or ICU admission, with a cut-off of 66.5. Yang et al. [22] identify IL-6/Ly ratio > 2.5 as an independent risk factor for in-hospital mortality. The novelty of our study consists in the study of a population composed exclusively of critical ICU patients and in the use of the combined immune-inflammatory index IL-6/Ly as a prognostic factor for mortality in this group.

Although the median values of ferritin and D-dimers exceeded the reference range in both survivors and non-survivors, we found no statistically significant difference among groups for these parameters. A number of studies identify serum ferritin as a predictor of in-hospital mortality [40,41]; however, the complex role of ferritin in COVID-19 inflammation has not been fully elucidated, as hyperferritinemia seems to be connected to the progression of infection, but could also act as a modulator in the process of COVID-19 pathogenesis [42]. Several reports have found elevated D-dimers in COVID-19 patients and suggest a correlation of D-dimers with poor clinical outcome [43,44]. A cause of our findings being inconsistent with previous studies is the static determination of parameters, instead of a more accurate dynamic evaluation.

AST was the only marker of hepatic function that demonstrated significantly higher values in non-survivors (*p* = 0.02), patients with AST > 44.15 U/L having a higher risk of in-hospital mortality. Bloom et al. also reported a pattern of aminotransferase elevation with the predominance of AST in COVID-19 patients, as opposed to the classical pattern of viral hepatitis characterized by more notable rises in ALT [45]. These findings could reflect the systemic effects of SARS-CoV-2 infection and suggest the possibility of COVID-19-induced mitochondrial dysfunction. Furthermore, creatinine and urea as indicators of kidney function were significantly increased in the non-survivor group (*p* = 0.03 and *p* = 0.0001, respectively) with cut-off values of 0.83 mg/dL for creatinine (sensitivity 57.83%; specificity 62.96%) and 55.85 mg/dL for urea (sensitivity 76.12%; specificity 76.19%). ROC curve analysis demonstrated a higher AUC for urea (AUC = 0.772) in comparison with creatinine (AUC = 0.638). As shown in Figure 1B, urea had the highest AUC among the investigated routine laboratory parameters. This particular finding could be explained by the intensity of the protein catabolism, which is reflected in the elevated values for urea.

Based on our results, INR constitutes a statistically significant parameter for in-hospital mortality of critical COVID-19 patients (*p* = 0.0056) with a cut-off level of 1.075 (sensitivity 67.09%, specificity 64% and AUC = 0.684). Another retrospective study reported a similar cut-off point for INR of 1.08 with higher sensitivity, specificity (sensitivity 70%; specificity 71%) and a greater AUC (AUC = 0.724) [46]. In a meta-analysis performed by Zinellu et al. [47], the prolonged INR is associated with COVID-19 mortality.

WBC is the only parameter of the complete blood count with a statistical significance on the patient survival identified in our analysis, with a cut-off value of 11.68 × 10^9^/L (AUC = 0.746, *p* = 0.02). Our findings are consistent with past reports, and a meta-analysis of 25 studies revealed WBC > 10 × 10^9^/L as a risk factor for COVID-19 progression [48].

In our study, patients with pH < 7.455 had a higher likelihood of in-hospital mortality (*p* = 0.0029), with other acid-base parameters such as lactate, pO2 and SO2 demonstrating no significant differences between survivors and non-survivors. These results should be carefully interpreted, as they are obtained for critical patients, under oxygen therapy and in the attempt to correct acid-base imbalances.

The only demographic parameter that presented significant differences between survivors and non-survivors in our analysis was the age of patients. This suggests the value of incorporating age alongside peripheral blood biomarkers in developing a risk scoring system for COVID-19 severity, in line with the findings of Lee et al. [49].

Our study presents several limitations, inherent to its retrospective design. In addition, the main limitations of our study consist in the relatively reduced number of critical patients meeting the inclusion criteria, having both clinical and laboratory data, including acid-base parameters and lack of data regarding vaccination status. Furthermore, the study was performed in a single center. The number of days from patient admission to determining IL-6 varied in the absence of a standardized testing protocol. For a more accurate prediction of clinical evolution, dynamic determinations of serum laboratory parameters at established time intervals would be preferable.

## 4. Materials and Methods

We conducted a single-center retrospective observational study in the Emergency County Hospital in Târgu Mureș, Romania between September 2020 and October 2021. The study was carried out with the approval of the Ethics Committee of the Emergency County Hospital Târgu Mureș, Romania (nr.Ad. 26973/10.11.2021). Eligible patients met the following inclusion criteria: (1) they had a confirmed COVID-19 diagnosis using reverse transcription polymerase chain reaction (RT-PCR) molecular testing; (2) they were hospitalized in the ICU; (3) they were adult patients (age range 29–88 years); (4) at least one IL-6 determination was ordered by the attending physician; (5) complete laboratory and clinical data were available. The impact of vaccination status on our study results was not evaluated due to the lack of availability of the vaccine for a considerable period of the time interval included in our retrospective analysis. A total number of 117 critical patients were included in the final analysis.

Clinical and laboratory data was retrieved from the electronic health records and from reviewing patient charts. We recorded demographic information (age, sex), past medical history, clinical symptoms, administered therapy and complications arising during hospitalization. Laboratory data included inflammatory markers: interleukin-6 (IL-6), C-reactive protein (CRP), ferritin, D-dimers; and inflammatory indices: interleukin-6 to lymphocyte ratio (IL-6/Ly), neutrophil to lymphocyte ratio (NLR) and systemic immune inflammation index (SII). Multi-organ damage was investigated by recording the results of biochemical tests (creatinine, urea, aspartate aminotransferase-AST, alanine aminotransferase-ALT, total serum bilirubin, conjugated bilirubin and blood glucose), coagulation tests (international normalized ratio-INR), complete blood count and acid-base markers (pH, PO2, SO2 anad lactate). Laboratory findings registered in our database were determined within 24 h of the IL-6 blood concentration measurement. The mean duration of time between the patient’s ICU admission date and the date of IL-6 testing was 2.39 days ± 2.29 days.

We performed a descriptive statistical analysis of all variables. The distribution of continuous variables was assessed using the Kolomogorov-Smirnov test. Parametric data was presented as mean ± standard deviation and non-parametric data as median and interquartile range. Comparison of data sets between survivors and non-survivors was performed using independent samples t-test or the non-parametric equivalent Mann-Whitney U test, when applicable. Categorical variables were expressed as frequency percentages and analyzed using Fisher’s exact test. Subsequently, optimal cut-off levels and AUCs (area under the curve) for statistically significant variables were determined using ROC (receiver operating characteristic) curves. The survival data was analyzed based on the Kaplan-Meier method and the log-rank test. We calculated two-sided *p*-values and considered *p*-values of <0.05 statistically significant. Statistical analysis was conducted using the GraphPad Prism 9.

## 5. Conclusions

Inflammatory parameters (IL-6, CRP; IL-6/Ly ratio) are significantly elevated in non-survivor critically-ill patients with COVID-19. Survival analysis demonstrated the association of IL-6 > 27.68 pg/mL, IL-6/Ly > 50.39, AST > 44.15 U/L, INR > 1.075 and pH < 7.455 with an in-hospital mortality for critical COVID-19 patients. The findings of our study highlight the importance of integrating inflammatory biomarkers with other routinely available laboratory tests in the prognostic assessment of critically ill COVID-19 patients. The confirmation of these results and further research in developing prognostic scoring systems based on laboratory parameters should be considered in future studies.

## Figures and Tables

**Table 1:** Comparison of demographic and clinical characteristics between survivors and non-survivors.

Variable	Non-Survivors (n = 89)	Survivors (n = 28)	*p*
Age (years)	66 ± 12	58 ± 14	0.0033 *
Male gender	46 (51.68%)	20 (71.42%)	0.0819 ^†^
Tocilizumab Pharmacotherapy	7 (7.86%)	1 (3.57%)	0.6779 ^†^
Remdesivir Pharmacotherapy	42 (47.19%)	16 (57.14%)	0.3927 ^†^
≥1 comorbidity (excluding type 2 diabetes)	82 (94.25%)	22 (78.57%)	0.0781 ^†^
Type 2 diabetes	38 (42.69%)	9 (32.14%)	0.3808 ^†^
Bacterial pulmonary infection	12 (13.48%)	1 (3.57%)	0.1855 ^†^

* independent samples *t*-test; ^†^ Fisher’s exact test.

**Table 2:** Comparison of laboratory characteristics between survivors and non-survivors.

Laboratory Parameter	Non-Survivors (n = 89)	Survivors (n = 28)	*p*
Inflammatory biomarkers			
IL-6 (pg/mL)	49.84 (20.85–107.6)	17.13 (7.468–37.43)	**0.0004 ^‡^**
Ferritin (ng/mL)	1357 (711.3–2392)	987.9 (588.2–2354)	0.1291 ^‡^
CRP (mg/L)	106.6 (67.39–174.4)	45.06 (23.81–119.6)	0.0270 ^‡^
D-Dimers (μg/mL)	1132.5 (529.5–5273)	735.25 (289–1138)	0.0857 ^‡^
Inflammatory indices			
IL-6/Ly	85.09 (22.74–163.3)	16.95 (8.44–60.42)	0.0003 ^‡^
NLR	14.47 (9.90–23.77)	12.19 (9.42–23.2)	0.0956 ^‡^
SII	3542 (2227–5582)	3083 (2089–5343)	0.3811 ^‡^
PLR	336 (256.8–491.7)	354.6(264.1–516.4)	0.9944 ^‡^
MLR	0.546 (0.443–0.814)	0.783 (0.442–1.283)	0.0587 ^‡^
Biochemical markers and coagulation tests			
AST (U/L)	54 (34.5–75.5)	33 (25–48)	0.0209 ^‡^
ALT (U/L)	40 (27–59.5)	53 (23–90)	0.4222 ^‡^
Total Bilirubin (mg/dL)	0.45 (0.32–0.62)	0.46 (0.36–0.67)	0.5763 ^‡^
Direct Bilirubin (mg/dL)	0.61 ± 1.03	0.32 ± 0.17	0.9062 *
Creatinine (mg/dL)	0.91 (0.72–1.81)	0.73 (0.65–1.04)	0.0306 ^‡^
Urea (mg/dL)	73.2 (56.1–133.8)	34 (28.35–73.5)	0.0002 ^‡^
Blood Glucose (mg/dL)	200.6 ± 73.05	192.1 ± 77.99	0.602 *
INR	1.12 (1.03–1.25)	1.05 (0.975–1.125)	0.0055 ^‡^
Complete blood count			
RBC (×10^6^/μL)	4.22 ± 0.63	4.45 ± 0.61	0.0934 *
Hemoglobin (g/dL)	12.23 ±1.99	12.97 ± 1.83	0.0919 *
Hematocrit (%)	37.57 ± 6.09	39.46 ± 5.07	0.1486 *
MCV (fL)	89.9 (86.85–94.5)	88.1 (86.39–92.1)	0.9134 ^‡^
MCH (pg)	29.3 (28.36–30.88)	29.02 (28–30.34)	0.4962 ^‡^
MCHC (g/dL)	32.52 ± 1.30	32.9 ± 1.41	0.2063 *
WBC (×10^9^/L)	12.73 (9.09–16.69)	9.33 (7.15–13.56)	0.0223 ^‡^
Neutrophils (×10^9^/L)	10.86 (7.558–14.02)	8.51 (5.88–12.13)	0.0596 ^‡^
Lymphocytes (×10^9^/L)	0.72 (0.42–0.99)	0.71 (0.47–0.92)	0.5967 ^‡^
Monocytes (×10^9^/L)	0.50 (0.30–0.83)	0.48 (0.3–0.59)	0.3295 ^‡^
Eosinophils (×10^9^/L)	0.006 (0–0.02)	0.005 (0–0.04)	0.9682 ^‡^
Basophils (×10^9^/L)	0.020 (0.008–0.044)	0.022 (0.011–0.035)	0.9692 ^‡^
Platelets (×10^3^/μL)	247.4 ± 104.2	267.6 ± 86.68	0.2411 *
Acid-base balance			
pH	7.44 (7.33–7.47)	7.47 (7.43–7.52)	0.0055 ^‡^
pO2 (mmHg)	72.55 (59.5–90.7)	73.5 (59.25–92.23)	0.9539 ^‡^
Lactate (mmol/L)	1.6 (1.1–2.1)	1.5 (1.2–2.1)	0.9654 ^‡^
SO2 (%)	92 (87.25–97)	94 (89–97)	0.8902 ^‡^

* independent samples *t*-test; ^‡^ Mann-Whitney U test. Values were expressed as mean ± SD for parametric data and as median (IQR-interquartile range) for nonparametric data.

**Table 3:** Receiver Operating Characteristic (ROC) analysis for statistically significant biomarkers in critically ill COVID-19 patients.

Laboratory Parameter	Cut-Off Value	AUC	95% CI	*p* Value	Sensitivity %	Specificity %
IL-6	27.68 pg/mL	0.721	0.61–0.833	0.0004	65.17	67.86
CRP	68.15 mg/L	0.689	0.527–0.851	0.027	76	66.67
IL-6/Ly	50.39	0.731	0.62–0.841	0.0003	60.49	70.37
Creatinine	0.83 mg/dL	0.638	0.518–0.758	0.031	57.83	62.96
Urea	55.85 mg/dL	0.772	0.631–0.91	0.0002	76.12	76.19
AST	44.15 U/L	0.66	0.53–0.79	0.0209	61.64	65.22
INR	1.075	0.684	0.564–0.805	0.0055	67.09	64
pH	7.455	0.676	0.565–0.787	0.0055	62.92	66.67
WBC	11.68 × 10^9^/L	0.647	0.527–0.766	0.0223	59.76	62.96

**Table 4:** Survival data analysis for critical COVID-19 patients.

Laboratory Parameter	Log-Rank Test *p* Value	HR	95% CI
IL-6	0.0002	2.17	1.43–3.29
CRP	0.1408	1.57	0.87–2.84
IL-6/Ly	0.0019	1.64	1.12–2.41
Creatinine	0.0521	1.36	0.93–1.98
Urea	<0.0001	2.22	1.45–3.38
AST	0.0094	1.56	1.04–2.33
INR	0.0091	1.54	1.05–2.26
pH	0.0029	0.62	0.43–0.9
WBC	0.2438	1.2	0.82–1.75

## Data Availability

The data presented in this study are available on request from the corresponding author. The data are not publicly available due to privacy restiction.
